# Increased Risk of Major Depression in the Three Years following a Femoral Neck Fracture–A National Population-Based Follow-Up Study

**DOI:** 10.1371/journal.pone.0089867

**Published:** 2014-03-13

**Authors:** Chih-Yu Chang, Wen-Liang Chen, Yi-Fan Liou, Chih-Chi Ke, Hua-Chin Lee, Hui-Ling Huang, Li-Ping Ciou, Chu-Chung Chou, Mei-Chueh Yang, Shinn-Ying Ho, Yan-Ren Lin

**Affiliations:** 1 Department of Emergency Medicine, Changhua Christian Hospital, Changhua, Taiwan; 2 Department of Biological Science and Technology, National Chiao Tung University, Hsinchu, Taiwan; 3 Institute of Bioinformatics and Systems Biology, National Chiao Tung University, Hsinchu, Taiwan; 4 Institute of Multimedia Engineering, National Chiao Tung University, Hsinchu, Taiwan; 5 School of Medicine, Chung Shan Medical University, Taichung, Taiwan; 6 School of Medicine, Taipei Medical University, Taipei, Taiwan; University of Southampton, United Kingdom

## Abstract

Femoral neck fracture is common in the elderly, and its impact has increased in aging societies. Comorbidities, poor levels of activity and pain may contribute to the development of depression, but these factors have not been well addressed. This study aims to investigate the frequency and risk of major depression after a femoral neck fracture using a nationwide population-based study. The Taiwan Longitudinal Health Insurance Database was used in this study. A total of 4,547 patients who were hospitalized for femoral neck fracture within 2003 to 2007 were recruited as a study group; 13,641 matched non-fracture participants were enrolled as a comparison group. Each patient was prospectively followed for 3 years to monitor the occurrence of major depression. Cox proportional-hazards models were used to compute the risk of major depression between members of the study and comparison group after adjusting for residence and socio-demographic characteristics. The most common physical comorbidities that were present after the fracture were also analyzed. The incidences of major depression were 1.2% (n = 55) and 0.7% (n = 95) in the study and comparison groups, respectively. The stratified Cox proportional analysis showed a covariate-adjusted hazard ratio of major depression among patients with femoral neck fracture that was 1.82 times greater (95% CI, 1.30–2.53) than that of the comparison group. Most major depressive episodes (34.5%) presented within the first 200 days following the fracture. In conclusion, patients with a femoral neck fracture are at an increased risk of subsequent major depression. Most importantly, major depressive episodes mainly occurred within the first 200 days following the fracture.

## Introduction

Femoral neck fracture is a common medical problem in patients over 60 years old. The incidence of this type of fracture is generally reported to be as high as 10%. [1]–[3] As the average life expectancy of the population increases, the number of femoral neck fractures increases each year; [4],[5] one previous study further predicted that the incidence of hip fracture will double by the year 2050. [6] Globally, the impact of femoral neck fracture has increased in a number of developed (or developing) countries, especially in those with large populations of aged individuals. [6] Physical complications after femoral neck fracture, including increased comorbidities (infections and cardiovascular diseases; 50%), poor self-care in functional and routine activities (40%) and acute or chronic pain (60%), have been reported. [7]–[10] Overall, high mortality rates are associated with hip fracture; recent studies have reported that the one-year mortality rate ranged from 14.7% (Korea) to 41.7% (Spain). [11]–[14] Acute stress and poorer quality of life are usually present in most femoral neck fracture patients. Indeed, one study reported that 50 to 75% of patients with femoral neck fracture never reach their previous functional levels [8].

A depressive episode is characterized by disturbances in major life areas, including mood, psychomotor activity, cognition, and vegetative function. Furthermore, specific risk factors for depression have been reported: female gender, social isolation, low socioeconomic status, comorbid medical conditions, uncontrolled pain and functional impairment. [15] Therefore, we suspect that femoral neck fracture patients may experience major depression after injury related to several risk factors during the post-fracture period. Some previous studies have reported that hip fracture could increase the chance of suffering depressive symptoms (including anxiety and insomnia) in patients with older age or poorer quality of life; [16], [17] however, no published study has focused on major depression. Unlike anxiety or insomnia, major depression presents with more severe psychosis symptoms, and the principles of diagnosis must establish, through rigorous criteria, clinical distress and psychosocial impairment. In this study, we used a nationwide Taiwanese population database to prospectively examine the relationship between femoral neck fracture and the risk of major depression in a 3-year follow-up period in an Asian society. In our study, a diagnosis of femoral neck fracture was a warning sign to screen for increased risk of an impending major depressive episode.

## Methods

### 2.1 Database

The Longitudinal Health Insurance Database (LHID) was used for this study; the LHID enables researchers to examine the use of medical services in Taiwan starting from the year 1995. The LHID is maintained by the Bureau of National Health Insurance and provided to scientists in Taiwan for research purposes. The Taiwanese government launched its National Health Insurance (NHI) program in 1995 to provide affordable health care for all people residing in Taiwan. As of 2007, over 98% of the Taiwanese population was enrolled in this program. The LHID contains original data collected from approximately one million people. The data used in this study were randomly sampled from data collected within 2003 to 2007. No significant differences were found in the gender and age distributions or average payroll-related insurance premiums between people in the LHID and all people served by the NHI program. The details of the generation of the LHID are published online by the Taiwan National Health Research Institutes.

### 2.2 Study Setting and Population

This is a prospective cohort study. During the period from January 1, 2003, to December 31, 2007, two patient groups were classified using the LHID, which included the study group (i.e., patients with femoral neck fracture) and the comparison group (i.e., patients without femoral neck fracture). The first hospitalization for treatment of femoral neck fracture during this period was coded as the index hospitalization. The comparison group’s index hospitalizations were set to be in the same month as those of the study patients. For control patients, the date that they were extracted was their index hospitalization. In this study, all patients were prospectively followed for 3 years after their index hospitalization. The probability of experiencing new-onset depression during the 3-year follow-up period was analyzed between the two groups.

### 2.3 Inclusion Criteria

#### Definition of patients with femoral neck fracture

We defined our group of femoral neck fracture patients as patients who were hospitalized with a principal diagnosis of femoral neck fracture according to criteria in the International Classification of Diseases, 9th Revision, Clinical Modification (ICD-9-CM; code 820).

#### Definition of patients with major depression

Major depression was defined as a principal diagnosis using ICD-9-CM criteria listed under codes 296.2, 296.3. The diagnosis of major depression adhered to the Diagnostic and Statistical Manual of Mental Disorders (DMS) - IV published by the American Psychiatric Association. The ICD-9-CM diagnosis of major depression was made by psychiatrists in this study.

### 2.4 Exclusion Criteria

All patients aged <18 years (n = 82) were excluded from this study. All patients who were previously diagnosed with femoral neck fracture or major depression prior to their index hospitalization were excluded from this study. Bipolar depression, substance-caused depression, dysthymic disorder and postpartum depression were not included. Moreover, affective disorder and depressive disorder were also not included because the principles of their diagnosis and their clinical presentations are different from those of major depression. Affective disorder may include manic attacks, and depressive disorder has only some of the same symptoms as major depression.

### 2.5 Quality Control for Potential ICD-9 over Coding and Treatments

To make sure the medical resource (budget) that provided by NHI program (government supported) would not be over used by the treating hospitals or patients; therefore, the diagnosis, treatments, medications of each patient was randomly and routinely inspected by specialists. Over treatment or over ICD-9 coding was not permitted and might result in fines.

### 2.6 Study Protocol

**Figure 1:**
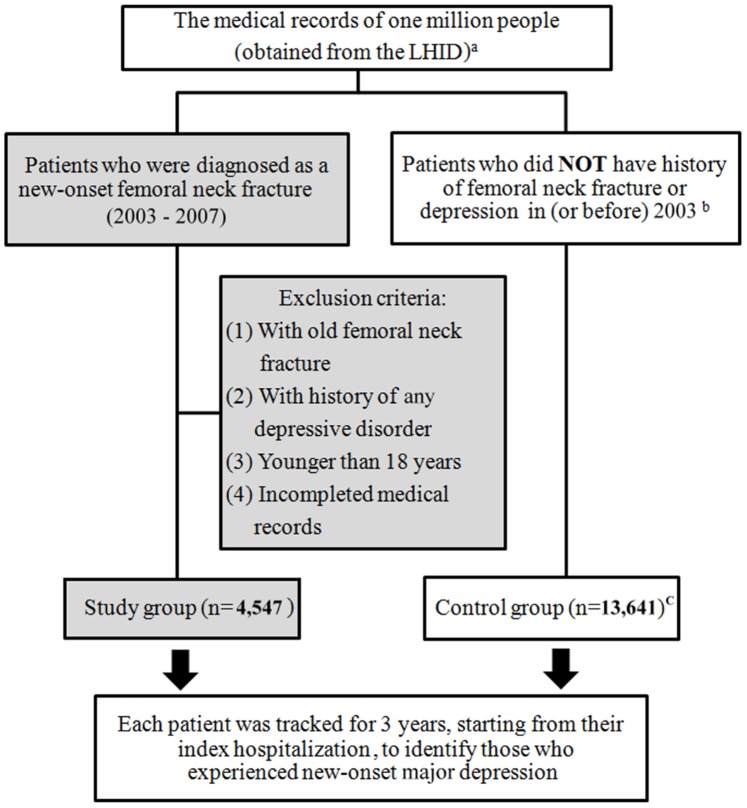
Flowchart of the selection methods in study and comparison patients. ^a^ The LHID contained medical records of one million people, which was randomly selected from the Taiwan National Health Insurance (NHI) program (supported by Taiwan government and over 98% of the Taiwanese population was enrolled in this program). ^b^ All personal medical records (diagnosis, treatments, medications), which had been recorded by different hospitals, were finally input into the NHI for requiring payments. Because almost all hospitals in Taiwan have joined the NHI; therefore, we could use it to screen patients’ past histories. ^C^ Three comparison patients for each femoral neck fracture patient (matched with study group patients in terms of sex, age and years of healthcare use).

Our study group included 4,547 patients with femoral neck fractures. The comparison group was selected from the remaining NHI beneficiaries registered in the LHID. We randomly selected 13,641 comparison patients (three comparison patients for each femoral neck fracture patient) who were matched with study group patients in terms of sex, age (<40, 40 to 49, 50 to 59, 60 to 69, 70 to 79, and >80 years) and years of healthcare use. As a result, a total of 18,118, patients were included in this study. The selection methods of study and comparison patients were summarized in Figure 1.

### 2.7 Data Analysis

The statistical package SAS was used for the analyses in this study. SAS was used to select the study and comparison groups (SAS Institute Inc., Cary, NC, USA). Descriptive analyses of the independent variables (patient characteristics, demographics, personal history at baseline, surgical interventions, the amount of time between fracture and the onset of depression and other comorbidities) are reported as percentages or the mean ± standard deviation (SD). The *X^2^* test was used to make between-groups comparisons of patient demographics, including economic status (monthly income: USD$>1000, USD$601∼1000, USD$<600), urbanization of their home city (level 1 to 4), the geographic location of patients’ residence (northern, central, southern, and eastern Taiwan), personal history at baseline (diabetes mellitus, hypertension, renal failure, liver cirrhosis, stroke and osteoporosis) and mortality rate between patients with fracture and non-fracture. The urbanization of patients’ home city was defined by population and certain indicators of the city’s level of development. Level 1 urbanization was defined as having a population greater than 1,250,000 people and a specific status of political, economic, cultural and metropolitan development. Level 2 urbanization was defined as having a population between 500,000 and 1,250,000 and an important role in the Taiwanese political system, economy and culture. Urbanization levels 3 and 4 were defined as having a population between 150,000 and 500,000 and less than 150,000, respectively. Furthermore, a crude hazard ratio (HR) was calculated using Cox’s stratified proportional hazards model (stratified with age, sex and the number of years since index hospitalization) to analyze the risk of new-onset major depression between the study and comparison groups. The covariate-adjusted HR was analyzed after adjusting for diabetes mellitus, hypertension, renal failure, liver cirrhosis, stroke, osteoporosis, geographic regions, post-fracture co-morbidities, monthly income and urbanization of patients’ home cities. In addition, we used SAS to analyze the eight most common post-fracture comorbidities during the study period. The relationship between post-fracture comorbidities and major depression was also analyzed. We used the Kaplan-Meier method and Log-rank test to estimate survival curves and compare the 3-year major depression-free survival rate between patients with femoral neck fracture and those without. Among the femoral neck fracture patients, the amount of time before the onset of major depression was recorded and divided into 6 periods (<200, 201–400, 401–600, 601–800, 801–1000 and >1000 days). Additionally, the relationship between surgical interventions (including hip replacement and open reduction of internal fixation of the hip) and the chance of suffering new-onset major depression was analyzed for this group (*X^2^* test). Hip replacement included hip arthroplasty in this study. A p-value <0.05 was considered to be statistically significant.

### 2.8 Ethics Statement

This study was exempt from a full review by the Institutional Review Board (permission code: 121007) because the data set consists of de-identified secondary data, which was released without restrictions for research purposes.

## Results

### 3.1 Demographic and Medical Treatment Data Collected from Patients with Femoral Neck Fracture

The characteristics and personal histories between patients with femoral neck fracture (n = 4,547) and those in the comparison group (without fracture; n = 13,641) are presented in Table 1. Females were the majority in both groups. Most femoral neck fractures occurred in the age groups of 70 to 79 (33.12%) and >80 years (33.91%). Compared with the control patients, femoral neck fracture patients had generally lower economic and urbanization levels. Fracture patients also had a higher prevalence of diseases in their personal medical histories at baseline compared with the non-fracture patients. These diseases included diabetes mellitus, renal failure, liver cirrhosis, stroke, and osteoporosis (for all of the above findings, *p*<0.05). The overall mortality rate during the study period was higher in patients with fractures (12.8%, n = 525) than in those without fractures (6.9%, n = 857) (*p*<0.001). Peptic ulcer (n = 1,644), chronic obstructive pulmonary disease (n = 1,547), pneumonia (n = 1,376), cellulitis (n = 1,041), heart failure (n = 896), arrhythmia (n = 832), stroke (n = 801) and deep vein thrombosis (n = 356) were the most common post-fracture comorbidities.

**Table 1:** Characteristics and personal histories between patients with femoral neck fracture and comparison patients.

	Patients with femoral neck fracture (n = 4,547)		Comparison patients (n = 13,641)		
	No.	%	No.	%	*p*-value
Gender					1.000
Male	2029	44.6	6087	44.6	
Female	2518	55.4	7554	55.4	
Mean age (y/o) (Mean±SD)	71.4±16.6		70.4±16.2		1.000
Age group (y/o)					1.000
18–39	324	7.1	972	7.1	
40–49	234	5.2	702	5.2	
50–59	324	7.1	972	7.1	
60–69	617	13.6	1851	13.6	
70–79	1506	33.1	4518	33.1	
> = 80	1542	33.9	4626	33.9	
Economic level (monthly income) (USD$)*					<0.001
<600	2744	60.4	7783	57.1	
601∼1000	1682	37.0	5167	37.9	
>1000	121	2.7	691	5.1	
Urbanization					<0.001
1 (most)	921	20.3	3207	23.5	
2	339	7.5	959	7.0	
3	1021	22.5	3003	22.0	
4	2266	49.9	6472	47.5	
Geographic regions of Taiwan*					0.004
Northern	2019	44.4	6380	46.8	
Central	900	19.8	2492	18.3	
Southern	1472	32.4	4389	32.2	
Eastern	156	3.4	380	2.8	
Personal history					
Diabetes Mellitus*	959	21.1	2619	19.2	0.006
Hypertension	1349	29.7	4038	29.6	0.941
Renal failure*	832	18.3	1491	11.0	<0.001
Liver cirrhosis*	223	4.9	418	3.1	<0.001
Stroke*	943	20.7	1809	13.3	<0.001
Osteoporosis*	1660	36.5	2237	16.4	<0.001

*Significant differences.

### 3.2 Depression Likelihood based on the Crude HR

During the 3-year follow-up period, we found that the risk of presenting with new-onset major depression was significantly higher in patients with femoral neck fracture than in comparison patients. In this study, 1.2% (n = 55) of patients experienced new-onset major depression after a femoral neck fracture, while the percentage of new-onset depression was only 0.7% (n = 95) in the comparison group. The stratified Cox proportional hazard analysis showed that the femoral neck fracture group had a crude HR 1.82 times greater than that of the comparison group (95% CI, 1.30–2.53; *p* = 0.001) (Table 2). After adjusting for patients’ geographic regions, monthly incomes, and personal histories at baseline, patients with femoral neck fracture were more likely to experience new-onset depression than were the comparison group patients (HR: 1.46, 95% CI: 1.03–2.08) (data not shown). Furthermore, the HR obviously increased to 3.54 (95% CI: 1.21–10.35) after considering the eight most common post-fracture comorbidities (Table 3). Among the 4,547 patients with femoral neck fracture, most of them received surgical interventions (n = 3,600, 78.2%). Open reduction of internal fixation was the most common surgical method (n = 2,392, 52.6%). Comparing with the non-operation patients (n = 947), the prevalence of depression did not significantly decrease in those who had received operation (*p* = 0.505).

**Table 2:** Crude HR for the presence of new-onset major depression among patients with femoral neck fracture and the comparison patients.

Presence of depression	Total sample (n = 18,188)		Patients with femoral neck fracture (n = 4,547)		Comparison patients (n = 13,641)	
3-year follow-up	No.	%	No.	%	No.	%
Yes	150	0.8	55	1.2	95	0.7
No	18,038	99.2	4,492	98.8	13,546	99.3
Crude HR (95% CI)	–		1.82* (1.30–2.53)		1.00	

*p- value = 0.001.

HR, hazard ratio.

**Table 3:** Covariate-adjusted HR for major depression during the 3-year follow-up period for major depression among the total patient sample (n = 18,118).

	Occurrence of new-onset depression		
Variables	HR	95% CI	*p*-value
**Groups**			
Patients with femoral neck fracture	3.54	1.21–10.35	0.021
Comparison*	1.00	1.00	1.000
**Personal history**			
Diabetes Mellitus**	1.90	1.02–3.53	0.044
Hypertension	0.78	0.40–1.51	0.459
Renal failure**	2.94	1.54–5.61	0.001
Liver cirrhosis	0.34	0.05–2.56	0.297
Stroke	0.49	0.19–1.25	0.135
Osteoporosis	1.16	0.63–2.15	0.638
**Cormobidity (post-fracture)**			
Cellulitis	0.99	0.52–1.87	0.970
Pneumonia	1.13	0.57–2.24	0.731
Chronic obstructive pulmonary disease	1.45	0.76–2.78	0.262
Arrhythmia	0.96	0.47–1.95	0.906
Heart failure	0.70	0.33–1.49	0.358
Deep vein thrombosis	1.64	0.74–3.63	0.222
Peptic ulcer	1.45	0.79–2.68	0.232
Stroke**	3.49	1.45–8.45	0.003
**Geographic regions of Taiwan**			
Northern*	1.00	1.00	1.000
Central	1.15	0.51–2.60	0.729
Southern	1.29	0.65–2.56	0.464
Eastern	0.88	0.31–2.46	0.803
**Economic level (monthly income) (USD$)**			
>1000*	1.00	1.00	1.000
601∼1000	0.51	0.11–2.47	0.406
<600	0.80	0.17–3.70	0.774
**Urbanization**			
1 (most)*	1.00	1.00	1.000
2	0.85	0.37–1.93	0.694
3	1.14	0.48–2.71	0.776
4	0.94	0.29–3.05	0.918

*Reference group.

** Higher risk of major depression.

HR, hazard ratio.

CI, confidence interval.

### 3.3 Major Depression-Free Survival Curves for Patients

**Figure 2:**
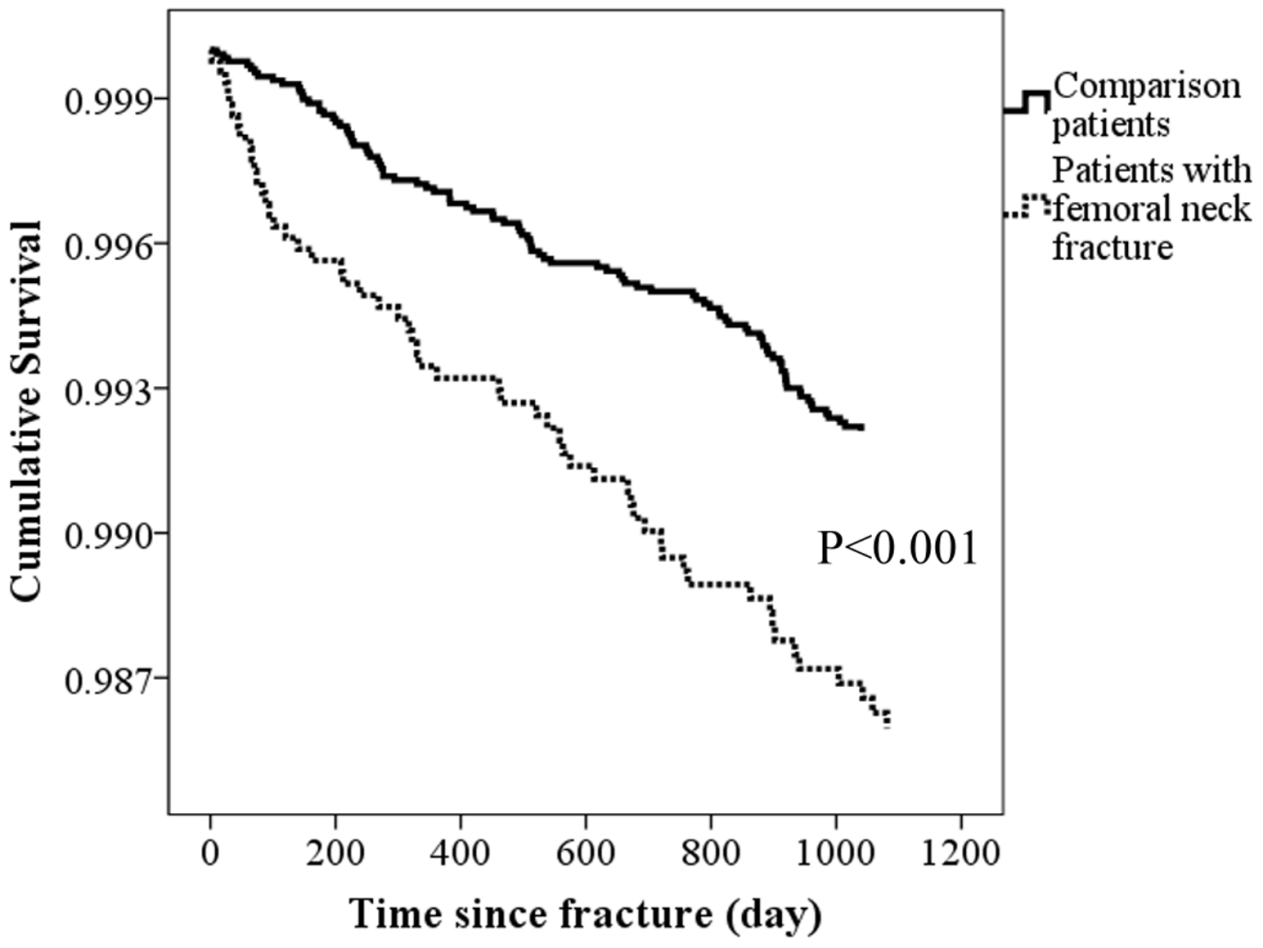
Time-related factor associated with the major depression occurrence. (A) Major depression-free survival curves for patients with femoral neck fracture and the comparison patients during the 3-year follow-up period (*p* = <0.001).

The major depression-free survival curves generated for the study period pertaining to femoral neck fracture patients and the comparison patients appear in Figure 2. We noted that patients with femoral neck fracture had significantly lower rates of 3-year major depression-free survival than the comparison patients did (*p*<0.001).

### 3.4 The Time between Fracture and the Onset of Depression

**Figure 3:**
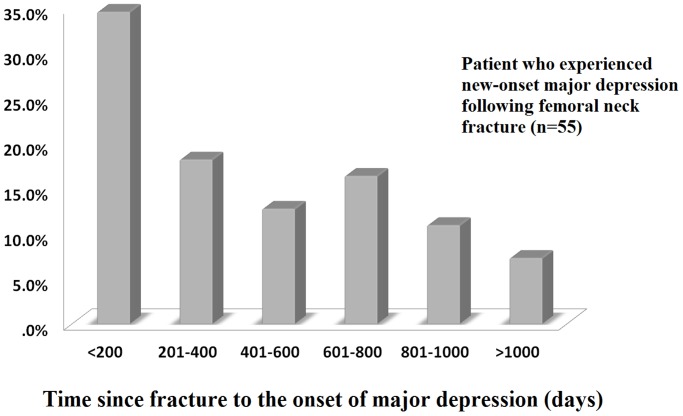
Most major depressive episodes (34.5%) occurred within the first 200 days following femoral neck fracture. The percentage of major depression patients also gradually decreased as the observation period prolonged.

The amount of time between fracture and the presentation of new-onset major depression during the study period is shown in Figure 3. Most major depressive episodes occurred within the first 200 days following fracture.

## Discussion

In this 3-year follow-up study, we found that femoral neck fracture was significantly related to the occurrence of new-onset major depression (1.82 times the rate among the control subjects without femoral neck fracture). After adjusting for patients’ demographic data, including personal histories at baseline, economic status and geographic regions, femoral neck fracture was associated with a statistically significant increased risk of major depression (HR:1.46). After the fracture, the condition of recovery might also influence patients’ emotions. Phillips AC et al. reported that poorer long-term recovery post-injury increased the chance of suffering depressive disorder; [16] however, post-fracture comorbidities were not well addressed. In this study, we noted that the HR of major depression obviously increased (1.46 to 3.54) after considering the comorbidities after femoral neck fracture. Among these, the adjusting analysis revealed that having a history of diabetes, renal failure or post-fracture stroke was the most powerful risk factor for major depression. The development of major depression is typically complex (including endocrine, gene, and immune activation), [18]–[20] and might not only relate to the risk factors that we have shown in this study.

Reduced quality of life and stress are usually present in femoral neck fracture patients. [15] It is well-known that life stress may be associated with depressive disorders. Some previous studies attempted to analyze elderly patients with hip fracture regarding cognitive impairment and mood disorder. These studies showed that 13 to 37.6% of the elderly had depressive mood or anxiety after hip fracture by self-report or score evaluation. [21]–[23] Compared with these studies, the incidence in this study was relatively low (1.2%) for three possible reasons. First, major depression has more serious diagnosis criteria than do depressive disorder or anxiety (in this study, bipolar depression, substance-caused depression, dysthymic disorder and postpartum depression were all excluded). Second, the diagnoses in this study were confirmed by a psychiatrist (not by self-report). Finally, our population not only focused on the elderly, and patients who had a history of depression were excluded before the study began.

Gambatesa M et al. analyzed 40 hip fracture patients for 30 days and found that their fracture-related anxiety scores did not obviously decrease, particularly among those who did not receive proper post-fracture education. [17] In this study, we further extended the observation period. Most major depressive episodes (34.5%) occurred within the first 200 days following a femoral neck fracture. The percentage of patients with major depression also gradually decreased as the observation period continued (<200 days: 34.5%; 201–400 days: 18.2%; 401–600 days: 12.7%; 601–800 days: 16.4%; 801–1000 days: 10.9%; >1000 days: 7.3%). Therefore, emotional support and depression prevention should be emphasized with primary physicians during the early stage of femoral neck fracture (early psychological intervention may be necessary).

Some studies have reported that surgical intervention for femoral neck fracture may improve quality of life. [8], In this study, 3,600 patients with femoral neck fracture underwent early surgical intervention (hip replacement and ORIF); only a small number, 46 (1.3%), of these patients experienced major depression. The percentage of surgical patients who experienced major depression was similar to that among the patients (n = 9; 1.0%) who did not undergo surgery. We suspect that life stress may persist after an operation because of increased medical costs (to pay for the operation) or the stress of wound care.

In conclusion, femoral neck fracture patients are at a high risk of experiencing subsequent major depression. More importantly, 34.5% of major depressive episodes occurred within the first 200 days following fracture. Early mental support or psychological intervention may be useful.

### 4.1 Limitation

The nature limitation of database study was the ICD-9 coding (which depending on the treating physicians). However, as the statements that we mentioned in the method section (quality control), the diagnosis and treatments of each patient were randomly and routinely inspected by specialists for controlling the government budget. For example, the payments for treating femoral neck fracture would be retrospectively inspected with patient’s medical records and image reports. In addition, a new-onset major depressive disorder was favor to be diagnosed with a detailed history taking, social worker’s records and psychiatrist’s consultations. Therefore, we suspected this nature limitation has been obviously decreased in this study. Because the LHID started in 1995, diagnoses made before 1995 were not included. This is a natural limitation for researchers who analyze data using the LHID database. [26], [27], [28] Therefore, we suspected that some patients had diagnoses of femoral neck fracture or depression before 1995, but they could not be clearly excluded. To decrease this limitation, patients who had any medical record of femoral neck fracture or major depression from 1995 to 2003 were excluded. Finally, because of some specific medications, psychological treatment and counseling are only provided by psychiatrists with the Taiwanese government’s permission. Therefore, nearly all patients who are suspected of initial major depression are referred to psychiatrists for complete evaluation. Therefore, we only included the psychiatrists’ diagnoses.
